# Miscellany

**Published:** 1911-09

**Authors:** 


### MISCELLANY

### The Medical Journal as an Advertising Medium

MANY business men, otherwise, experienced and shrewd, fail to appreciate the full value of medical journal advertising. There are probably not more than three or four medical journals in the world whose circulation even approximates that of the average, successful, literary magazine. But the limited circulation of the medical journal is sifted. It does not include children, it eliminates the adults who read for amusement, it is confined to responsible, grown men, mostly heads of families, practically all centers of influence and of advice with regard to the dwelling, clothing, lighting, heating, food and other necessities and comforts of life used by the community at large.

While few physicians are rich, the profession, on the average represents the upper 15 per cent. of the population, in the earning scale and, on the average, its necessary expenditures are disproportionately high compared with its earnings. That is to say, the advertiser in a medical journal eliminates the 85 per cent. of the population which cannot, except by an unwarrantable extravagance, buy any kind of luxury and which cannot buy the higher grades of necessities. Moreover, the average grade of any necessity or moderate luxury, bought by the physician is higher than that bought by the upper 15 per cent. of the population and corresponds to that which about 5 per cent. of the grown men who buy ordinary magazines can afford to purchase.

Another point to be considered is this: There is no reason why the buyer of an ordinary magazine should read the advertisements and the buyers of magazines who are most likely to become buyers of goods, are least likely to have time to read the advertisements. On the other hand, the physician who reads a medical journal at all, simply must give careful consideration to its advertisements of drugs, instruments, laboratories, and the like, if he gets any practical benefit from his subscription. In this respect, the medical journal is like a trade journal, only "more so."

---

### Prize Essay

Under the auspices of The American Society for the Study of Alcohol and Other Narcotics.

Dr. L. D. Mason, of Brooklyn, N. Y., Vice-President of the society, offers a prize of $150.00 for the best essay on the following topic:

*"The Biological and Physiological Relations of Alcohol to Life."*

The essay must be the result of original research which shall confirm or disprove the present theories of the inherited effects of alcoholic degeneration and indicate how far the defects of parents are transmitted to the children.

Such work may be carried on in man or animals, and the results may be illustrated by drawings or photographs and must be typewritten and sent to the office of the Secretary before July 1, 1913.

This offer is open to students in all countries, and each essay should be accompanied by a motto and a sealed envelope containing the same, with the author's name and address.

THE chairman of the committee of award is Dr. W. S. Hall, Professor of Physiology in the Northwestern University, Chicago, Ill.

All inquiries should be addressed to Dr. T. D. Crothers, Hartford, Connecticut, Secretary.

---

ON THE MECHANISM OF THE PRESYSTOLIC MURMUR.—Stuart Hart, New York, from a careful study of four cases of presystolic murmurs, believes that in certain cases the short crescendo murmur preceding the first sound of the heart in mitral stenosis is not due to auricular activity. The four cases are described in detail. They presented well marked clinical designs of mitral stenosis. There was at no time an absence of the presystolic thrill and murmur in any of the cases. The graphic records of these showed the ventricular form of venous pulse and an entire absence of evidence of gross auricular contraction.—*Medical Record,* July 1, 1911.

---

A CLINICAL DEMONSTRATION OF THE FATALITY OF TEXTBOOK HYDROTHERAPY—A WARNING.—Simon Baruch, New York, makes a plea for a more rational use of hydrotherapy. The average textbook gives directions for cold baths which if followed out will cause fatality in cases of typhoid fever and heat stroke. The author has given in his book directions for the practical use of baths, and states that if these directions are carefully followed they will give excellent results. Plunging an individual suffering from heat stroke into an ice bath will often prove fatal if the heart is weak, while a bath at faucet temperature will frequently result in saving life.—*Medical Record,* July 1, 1911.

---

THE MEDICAL ASPECTS OF MODERN LIFE INSURANCE.—Frederick Hoffman, Newark, N. J., states that insurance medicine is far behind the times as compared with general practice. There is no special insurance medical journal, and all articles on this subject must be published in the general medical press. These articles represent every branch and subject of insurance. Students of general medicine might well give attention to more rigid and careful examinations for insurance and for recruiting purposes.—*Medical Record,* July 1, 1911.

